# *Actinobacillus pleuropneumoniae*, surface proteins and virulence: a review

**DOI:** 10.3389/fvets.2023.1276712

**Published:** 2023-11-30

**Authors:** María M. Soto Perezchica, Alma L. Guerrero Barrera, Francisco J. Avelar Gonzalez, Teodulo Quezada Tristan, Osvaldo Macias Marin

**Affiliations:** ^1^Laboratorio de Biología Celular y Tisular, Departamento de Morfología, Centro de Ciencias Básicas, Universidad Autónoma de Aguascalientes, Aguascalientes, Mexico; ^2^Laboratorio de Estudios Ambientales, Departamento de Fisiología y Farmacología, Centro de Ciencias Básicas, Universidad Autónoma de Aguascalientes, Aguascalientes, Mexico; ^3^Departamento de Ciencias Veterinaria, Centro de Ciencias Agropecuarias, Universidad Autónoma de Aguascalientes, Aguascalientes, Mexico

**Keywords:** *Actinobacillus pleuropneumoniae*, bacterial envelope, membrane proteins, proteome, vaccine, biofilm

## Abstract

*Actinobacillus pleuropneumoniae* (App) is a globally distributed Gram-negative bacterium that produces porcine pleuropneumonia. This highly contagious disease produces high morbidity and mortality in the swine industry. However, no effective vaccine exists to prevent it. The infection caused by App provokes characteristic lesions, such as edema, inflammation, hemorrhage, and necrosis, that involve different virulence factors. The colonization and invasion of host surfaces involved structures and proteins such as outer membrane vesicles (OMVs), pili, flagella, adhesins, outer membrane proteins (OMPs), also participates proteases, autotransporters, and lipoproteins. The recent findings on surface structures and proteins described in this review highlight them as potential immunogens for vaccine development.

## Introduction

Porcine pleuropneumonia, caused by *Actinobacillus pleuropneumoniae* (App), is one of the respiratory diseases with the greatest economic impact worldwide (1). This disease is the source of great economic losses because it produces high morbidity, mortality, and costs for treating infected animals. Despite the clinical and sanitary management carried out within pig farms, the transmission of the disease is very easy to spread due to the portability of the bacteria from animals that survived the infection or that were asymptomatic (2–4).

To date, 19 serotypes have been recognized. The classification of biovarieties by their growth *in vitro* is of two types. Biovar 1, requires nicotinamide adenine dinucleotide (NAD) exogenously for growth, and Biovar 2, which is independent of NAD. Generally, serotypes 1 to 12 and 15 to 19 are biovar 1, and serotypes 13 and 14 are biovar 2 (1, 5, 6).

The prevalence of serotypes worldwide is very varied (7–19), making the infection very difficult to control since vaccines developed for some regions are not effective for others (9) (Table 1).

**Table 1:** Predominant serotypes of *Actinobacillus pleuropneumoniae* in the world.

Continent	Country	Predominant serotype	Another serotype	Reference
Asian	South Korea	1 and 5	2, 4, 7, 10 and 12	Lee et al. (7)
	Japan	2	6 and 15	Ozawa et al. (9)
	China	1, 3, 4, 5 and 7	-	Xu et al. (8)
Europe	Belgium	2	3, 9 and 5	Dom et al. (10)
	Hungary	2	13, 8 and 16	Sárközi et al. (11)
	Denmark	2	5, 6	Jessing et al. (14)
	England	8	2, 3, 6, 7 and 12	Li et al. (13)
Oceania	Australia	15	1, 5, 7 and 12	Turni et al. (12)
America	Canada and USA	5 and 7	1, 2, 6, 8, 13, 12, 15 and 17	Gottshalk et al. (15) and Lacouture et al. (18)
	Mexico	1	3, 4, 5, 7, 8	Ontiveros et al. (16), Serrano-Rubio et al. (17), and Williams et al. (19)

Transmission of App was thought to be solely due to direct contact of mucus and aerosol from one pig to another, but it has now been shown that App may be viable in water samples from pig farms, suggesting that it could function as a reservoir, Inoculum or vehicle to transmit the bacteria (20). Its viability in nasal secretions outside the pig is 3–4 days; it can survive at −20°C for 17 weeks and is maintained at −70°C in prolonged storage (21).

The App bacterium is usually considered a strict pathogen of the respiratory tract of pigs. However, there are scattered cases of it being detected as a causative agent in cases of arthritis, osteomyelitis, hepatitis, meningitis, and nephritis (22). The disease can range from hyperacute to chronic, depending on the number of bacteria that reach the lung. The main signs are fever, increased respiratory rate, coughing, sneezing, dyspnea, anorexia, ataxia, vomiting, diarrhea, and respiratory distress. The primary lesions occur at the level of the lungs, finding edema, inflammation, hemorrhage, and necrosis. In some cases, we can find hemorrhagic foam in the nose or mouth of animals that die. We can also find bloody fluid and fibrin in the thoracic cavity (23).

For App to install itself, persist in the host, and cause the characteristic lesions of the infection, it uses virulence factors. At present, bacterial virulence factors can be divided according to their mechanism of action: factors that involve adhesion to the host, factors in the acquisition of essential nutrients, factors involved in the induction of lesions, and the persistence of the infection (3). The bacterial membrane is composed of 50% of surface proteins, which participate diversely in virulence factors (24).

Due to the worldwide distribution of the serotypes, the high mortality and morbidity, the persistence in infected animals, the easy transmission of the infection due to the survival of the bacteria in the environment, its antigenic variability, including virulence factors, it has been difficult to find a vaccine that manages to control and eradicate the presentation of the infection caused by App within pig farms. This is why alternatives for potential immunogens must be sought to develop new vaccines. In this review article, a global analysis of the structures and surface proteins, general characteristics, transport, and secretion is carried out. It lists surface proteins described to date and how they intervene in the virulence factors and a review of potential vaccines based on these proteins. Finally, techniques for the isolation and identification of a variety of proteins are classified, allowing the establishment of protocols that support the advancement of knowledge of the pathogenesis of the infection caused by App.

## Bacterial envelope and proteins transport

App bacterium is a Gram-negative, with typical coccobacillus morphology, hemolytic, urease positive, positive for mannitol, xylose, ribose, and lactose fermentation, and negative for glycerol, indole, and esculin (4, 25), of an approximate size of 0.4 × 1.0 μm, for its growth *in vitro,* requires aerobic or anaerobic conditions at a temperature of 37° C for a period of 24 to 72 h, as morphological characteristics the colonies are partially transparent, circular smooth, shiny and convex (26).

**Figure 1:**
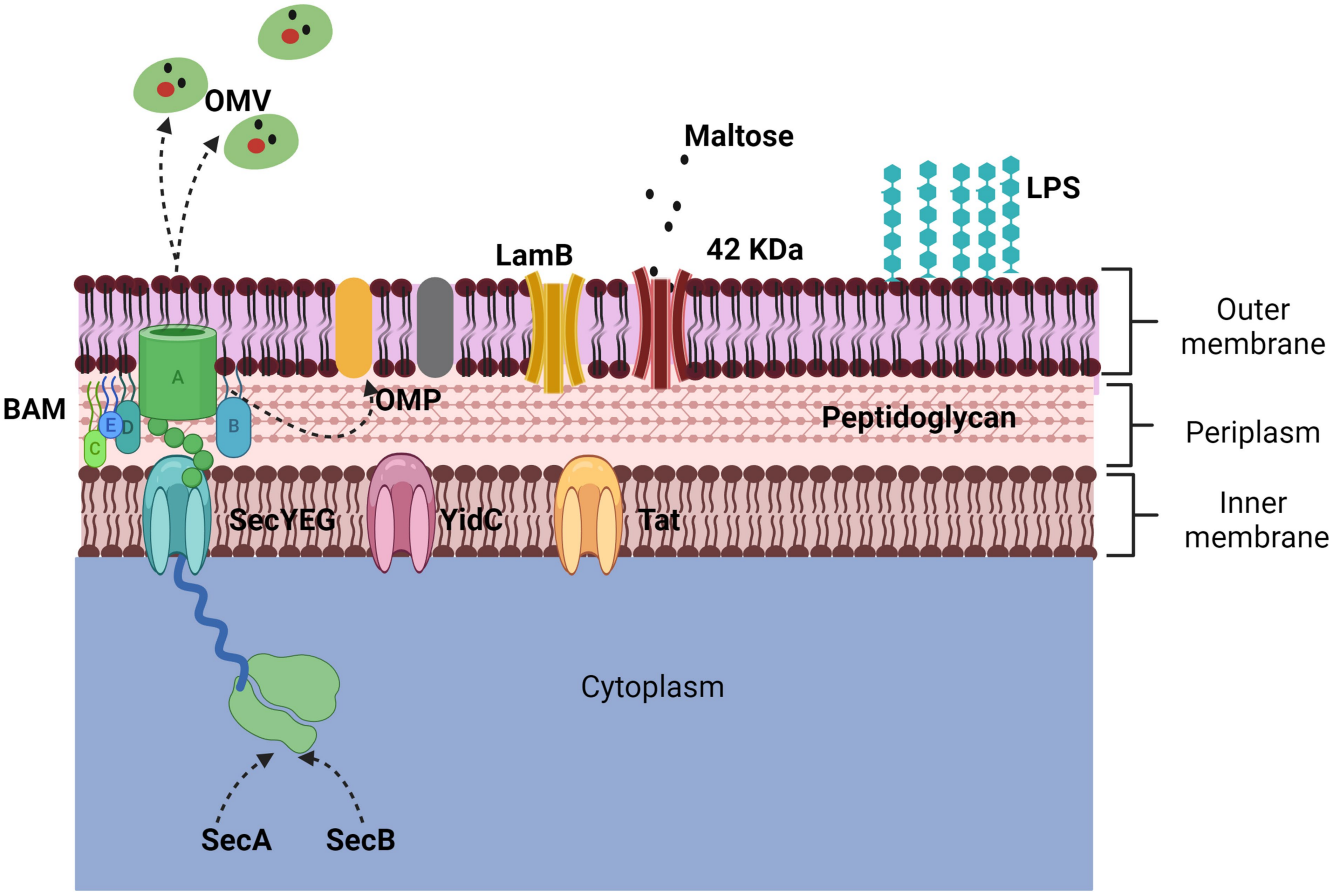
Representation of the outer membrane. The bacterial membrane is composed of a bilayer, the inner membrane, and the outer membrane. The two main proteins that make it up are porins and outer membrane proteins (OMPs). The 42 KDa protein identified in App is porin-like and is expressed in the presence of maltose while the LamB porin aids in antimicrobial resistance. The secretion of OMPs is regulated by three transporters (Sec, YidC, and Tad) through the BAM complex. The secretion mechanism of OMVs is not defined but they harbor numerous proteins and toxins.

The membrane of Gram-negative bacteria is composed of an inner and an outer membrane; between the two membranes, there is a peptidoglycan mesh that gives it shape and rigidity. The outer membrane is a phospholipid bilayer composed of many proteins, surface structures, and lipopolysaccharides (LPS); the inner membrane is in contact with newly synthesized proteins, where some are transported to the extracellular medium (27–29) (Figure 1).

One of the main proteins on the cell surface is the outer membrane proteins (OMPs), which can only be found in Gram-negative bacteria, mitochondria, and chloroplasts. The OMPs are composed of a β barrel fold, with an approximate size of 8 to 26 strands, and each protein is synthesized in the cytoplasm for a location and environment of the outer membrane (24, 30). Trafficking to the outer membrane begins with the secretion of their proteins, which are synthesized in the cytoplasmic ribosome, crossing the inner membrane. The inner membrane contains three types of transporters, Sec translocon, the YidC insertase, and the Tat system (31). The Sec translocon (SecYEG), through a small pore in the center, remains unfolded using chaperones (SecA and SecB), which emit a signal peptide to be delivered to the BAM complex for refolding and insertion into the outer membrane. However, it is still unknown in detail how this process happens; it is known that in some cases, the synthesis of some proteins is strongly induced when they are needed (Figure 1) (24, 28).

Most of the proteins transported by the Sec and Tat systems remain in the cell, either in the periplasm or on the inner membrane; some proteins can be transported out of the cell with the help of another secretion system, such as the In the case of exoproteins that use the Sec and Tat-dependent system and ultimately use systems that are based on the β-barrel channel that forms a ring in the outer membrane (Type I, II, III, and IV systems) (32).

On the other hand, other structures are also secreted by bacteria, which contain many proteins. Outer membrane vesicles (OMVs) are structures that derive from the envelopment of the bacterium (33), and were first described in Gram-negative bacteria, specifically in *Escherichia coli,* and subsequently in all Gram-negative bacteria related so far. OMVs are spherical particles that vary from 20 to 300 nm in size; the production mechanism of these vesicles is not yet fully elucidated (34).

## Adhesion proteins

Once the bacterium enters the host, the latter activates different defense mechanisms, such as mucus secretion, mucociliary action, and the use of immune system components to prevent adherence. On the contrary, the bacterium uses mechanisms that allow it to evade the host’s response and will enable it to remain inside it. Adherence of bacteria to the host mucosal epithelium is the first step in colonization and establishing pathogenesis. The adhesion of App to the tracheal cells of the porcine respiratory tract has been mainly associated with the lipopolysaccharides (LPS) present in the cell membrane of the different serotypes of this bacterium (35) mechanisms allow them to adhere firmly to the host mucosa are pili, flagella, fimbriae and specific adhesins (36, 37).

The pili are filamentous structures typical of Gram-negative bacteria and are composed of large biopolymers in the form of helices attached to the outer membrane (38, 39). Pili types and functions will vary depending on the type of bacteria; for example, type IV pili have a sliding motility function, while type 1 pili and P-pili from uropathogenic *Escherichia coli* and CS pili from enterotoxigenic *E. coli* have a function adhesion (40). The pili in App is a pili IV type (Tfp) or IVb type (Flp), which is formed in defined chemical media; in addition, this pili is essential for adherence and biofilm formation (41, 42). In interactions with epithelial cells, it has been shown that the Tfp protein is upregulated after adhesion has occurred (43).

In the case of adhesins that enhance surface binding for App is the ApfA adhesin, which has been suggested as an important virulence factor for the ability to colonize the lung (44). Adherence of the bacteria to alveolar epithelial cells has been demonstrated *in vitro* associated with the expression of fimbrial proteins and OMPs of 55 kDa, a protein not yet described (45) has also been proposed that a 60 kDa protein could be related to adhesion to extracellular matrix components such as collagen and fibrinogen (46).

Trimeric autotransporter adhesins (TAAs) are a family of proteins that are secreted by the outer membrane of Gram-negative bacteria; their structure comprises an N-terminal “passenger” domain followed by a C-terminal translocation unit which functions as an anchor of the membrane. These proteins have been identified in various pathogenic bacteria, attributing to them diverse functions such as adhesion, hemagglutination, and serum resistance (47). A few TAAs have been identified in App; the adhesin Adh the YadA-like head region of Apa1 has been identified as the optimal adhesion *in vitro* adhesion assays, in addition to regulating auto-agglutination, biofilm formation, and maintenance of bacterial cell morphology (48), Apa2H1 is the primary domain of the trimeric autotransporter adhesin Apa2 which has been shown to have an effect of activating an immune response and protecting against infection by App (49).

To prevent the adhesion of the bacteria to the host and stop the onset of infections, alternative vaccines have been chosen using pili, fimbriae, and adhesins. Although in App, the results of vaccines with fimbrial antigens and adhesins have only induced a strong and non-protective immune response, other studies against various bacterial pathogens such as *Moraxella bovis*, *Dichelobacter nodosus*, *Proteus mirabilis* y *E. coli* enterotoxigenic have been successful (50). Conducting trials with vaccines made with these proteins and others could provide a more significant immune response against App. A clear example of success was a vaccine with a chimeric protein called Ap97, which comprises a deletion derivative of the N-terminal region of the ApxIII toxin from App and the R1 and R2 repeats of the P97 adhesin from *Mycoplasma hyopneumoniae* P97, which conferred protection to challenged pigs against these pathogens (51). Vaccines made with adhesins allow the stimulation of the protection of protective antibodies, allowing the neutralization of the bacteria, inhibiting the attachment to the host tissue, and, through opsonization, causing microbial death by phagocytosis or complement activity (52).

## Nutrients acquisition

Bacteria need nutritional requirements such as sugars, lipids, proteins, peptides, oligonucleotides, xenobiotics, vitamins, minerals, and inorganic elements to survive, and the main routes for obtaining them are through anabolism and catabolism depending on the resources found around them.

One of the main cellular structures that function as transporters is the so-called porins, which, as their name suggests, form a pore in the bacterium’s outer membrane (53). Porins are now known to play an important role in energy production, photosynthesis, and nutrient transport (54). The OmpC and OmpF porins in *E. coli* were the first to be identified in Gram-negative bacteria; later, they were identified in other bacteria, including Gram-positive bacteria. Its structure can be trimeric or monomeric, which will depend on its physicochemical properties, its conformation, and the environment; for example, some Gram-negative bacteria, in case of nutrient limitation, adapt their permeability through the expression of OmpF and LamB porins, which are important for the absorption of sugar (55). Porins were described by Nakab in 1975 in *S. typhimurium* (56).

In App serotype 1, it has been possible to identify a 42 kDa protein expressed in growth conditions with maltose. This protein was described as a porin-type protein because it is associated with peptidoglycan and can resist trypsin proteolysis. Other maltose-inducible proteins have been described in *S. typhimurium* and *Vibrio cholerae* (57).

A study conducted by Ma et al. (58) found 12 porins in a strain of App (SC1810) and, through a transcriptome analysis, found that genes coding for the LamB and OmpP2B porins were downregulated, deletion of LamB led to a decrease in sensitivity to carbapenem drugs.

There is currently a commercial vaccine with a 42 kDa protein in combination with Apx toxins (Porcilis APP from MSD Animal Health and Pleurostar APP from Novartis Animal Health Canada Inc.); this vaccine has obtained favorable results as it reduces the prevalence of symptoms and respiratory lesions, in addition to reducing mortality and the use of antimicrobials (59). This reduction in the use of drugs could be precisely because porins have been related to this resistance to antimicrobials. One of the most impactful benefits of continuing to carry out this type of vaccine is that it helps prevent the establishment of infections with resistant strains in immunized individuals and reduces the resistance in other microorganisms by reducing the use of antibiotics (60).

Iron is another essential factor for growth, metabolism, and bacterial colonization in Gram-negative bacteria. It is also an indispensable factor in eukaryotic cells, leading to competition for this nutritional factor (61). When the bacterium is in contact with the host, it activates an immune response called nutritional immunity, which limits the production of iron and other metals to prevent absorption by pathogenic organisms (62). Several proteins in App contribute to the host’s defense of this immunity mechanism, which acts as virulence factors regarding iron acquisition.

The FhuA lipoprotein is a 75 kDa protein that acts as a ferrichrome receptor (63) is expressed *in vivo* (64) and is in all App serotypes (65). This protein encodes the receptor for ferrichrome TbpA/TbpB, of 110 and 60 kDa, respectively, lipoproteins related to transferrin binding (63) and is also associated with three other proteins FhuD (35.6 kDa), FhuC (28.5 kDa) and FhuB (69.4 kDa) proteins related to the translocation and transport of ferric hydroxamate (66). The FhuA protein has also been described in other bacteria (67), such as *E. coli*. It is attributed an important role not only in the transport of ferrichrome, but it can also become a high conductivity channel by binding to phage T5, T1, phi 80, colicin M and the antibiotic albomycin (68) so that it functions as a mediator for the transport of phage DNA (69) while in *Salmonella enterica* and *Salmonella bongori* it participates as a receptor for several bacteriophages and toxins (70).

The TbpA and TbpB proteins form a bipartite transferrin receptor; Tpbs are host-specific proteins and, therefore, will only bind transferrin from their host (71). The bacterium App expresses these proteins under iron-restricted conditions, which allows it to use porcine transferrin as the only source of iron and play an important role as a virulence factor during infection (72). Both proteins have also been found exposed on the surface of *Neisseria meningitidis,* forming a single complex (73) *Haemophilus (Glässerella) parasuis*. An increase in the genes of these two proteins has also been detected when they are under iron restriction stress (74).

The HgbA protein weighs approximately 105 kDa, is required for App growth on hemoglobin as the sole source of iron (64), and, like FhuA, is conserved in all App serotypes (65). This protein is essential in obtaining iron from other pathogenic microorganisms, such as *Haemophilus ducreyi*. The HgbA protein presents homology with HutA of *V. cholerae*, Tbp1, and Lbp1 of *Neisseria* spp., which also function as receptors for heme, transferrin, and lactoferrin groups (75).

The protective efficacy that the proteins involved with the iron acquisition factor in App can produce has been verified. A vaccine made with bacterial lysates containing the proteins TbpA, TbpB, HgbA, other membrane proteins, and exoproteins resulted in a humoral and protective response against serotypes 2 and 9 (76). There are no recent studies that evaluate the effectiveness of these proteins in App, but there are in other bacteria such as *H. parasuis* (74), *N. meningitidis* (77), and *Salmonella* spp. (78). The lack of up-to-date studies using these proteins is essential since reverse vaccinology studies suggest they could be potential immunogens for developing effective vaccines (67, 79). It has also been observed that the combination of some of these proteins involved in acquiring iron with others usually has satisfactory results in protecting against pathogenic infections (78, 80, 81).

## Induction of injuries and evasion of defense mechanisms

Pathogenic bacteria also use virulence factors to cause injury and thus ensure their permanence within the host. Bacterial species combine virulence factors to induce disease, and depending on the combinations, they can cause characteristic lesions; for example, enteropathogenic *E. coli* (EPEC) destroys intestinal microvilli, which induce infection and the production of diarrhea (38).

In App, there are characteristic pulmonary lesions that are caused by pore-forming toxins (PFT) called Apx toxins, which allow dissemination and colonization within the host; their mechanism will enable them to embed themselves in the membrane to perforate it later, causing an ionic imbalance and nutritional (82) having proteolytic, cytotoxic and edemogenic effect (83, 84).

To date, the 19 known serovar (5) produce four toxins in different ways (ApxI, ApxII, ApxIII, and ApxIV) (84). The ApxI, ApxII, and ApxIII toxins have a cytotoxic and hemolytic effect and, when released, can cause lysis of epithelial cells, alveolar cells, endothelial cells, red blood cells, neutrophils, and macrophages even in the presence of neutralizing antibodies, where the serotype relates the production of these toxins and not by the App strains (3, 85), the ApxIV toxin is expressed in infected pigs and not *in vitro* and is a determinant to confirm the infection caused by App (86).

The ApxI, Apx II, and ApxIII toxins have been widely cataloged with a rather important role as a virulence factor of App; it has been shown that they are responsible for the presence of respiratory symptoms in addition to the typical lesions of porcine pleuropneumonia both in endobronchial inoculations as in the analysis of tissues with necrosis of clinically diseased animals (87, 88). The ApxI toxin secreted by serotypes 1, 5, 9, and 11 is the most virulent due to the cytolysis and hemolysis it causes in experimental models (84, 89).

In the case of respiratory tract invasion by infectious agents, as the primary defense, the immune system responds with the activation of alveolar macrophages and neutrophils. It has been verified that even in the presence of antibodies against Apx toxins, App can destroy alveolar macrophages, unlike neutrophils protected by these antibodies (90). Specifically, ApxI can induce necrosis, apoptosis in porcine alveolar macrophages (PAM) (91), and pyroptosis by caspase-1-dependent cell death (92). The use of transposon mutagenesis in different animals has shown that Apx toxins are the main virulence factors of the infection caused by App (93).

Within the group of toxins of the RTX family, other exotoxins have also been identified in bacteria such as *Aggregatibacter actinomycetemcomitans* (LtxA), *Mannheimia haemolytica* (LktA), *Bordetella pertussis* (CyaA), uropathogenic *E. coli* (HlyA) where their central receptors are β2 integrins found in leukocytes (94).

The App bacterium has a great diversity of serotypes, and, as we have already mentioned, they secrete a variety of Apx toxins diversely. This antigenic variability has not allowed the development of a single vaccine for the various existing App serotypes. The investigation of the different virulence factors of App has focused on the development of vaccines that allow the creation of optimal immunity for the other serotypes of this bacterium, the most studied being the development of vaccines with Apx toxins. However, they have also developed polysaccharide and liposaccharide vaccine conjugates involving OMPs combined with hemolysin and OMPs (95).

There are other proteins involved with the induction of injury. VacJ was first described as an outer membrane lipoprotein of the bacterium *Shigella flexneri*, with a molecular weight of 28 kDa, as a protein capable of allowing intercellular dissemination and causing a protrusion in infected cells (96). Roier et al. (97) demonstrated that, as a virulence factor, the VacJ lipoprotein of the bacteria *Haemophilus influenzae* and *V. cholerae* contributes to the increase in OMVs, obtaining the same result Antenucci et al. (98), who was confirming its presence in App serotypes 3, 5b and 7, in addition to demonstrating that VacJ is highly conserved and expressed in App during natural infection. It has been verified that the OMVs also contain toxins, proteases (99), and antigenic proteins, which are attributed to the damping of the response of alveolar macrophages at the beginning of the infection by App (91).

Proteases allow bacteria to degrade misfolded proteins, act as regulators of their environment, and actively participate as virulence factors (100). Within this group of proteins, the HtrA protein, also initially described in *E. coli*, has been described in App as a serine protease that allows quality control of other proteins and is active in response to secretion stress in animal models. It has been proven to induce an immune response through the secretion of IgG, IFN-γ, and IL-2 and activates the proliferation of lymphocytes in the spleen (101).

In the past, it was believed that infectious diseases were controlled by discovering chemotherapeutic agents. However, this was not the case for toxin-producing infections where vaccination has not met expectations (102), as with infection by App. There are currently commercial vaccines made from toxins such as Porcilis APP (MSD Animal Health), Ingelvac® APPX (Boehringer Ingelheim), and Coglapix® (Ceva), but none have managed to control infection by App (95). Therefore, it is necessary to continue looking for alternative immunogens to develop new vaccines.

Reverse vaccinology analyses have also made it possible to establish the VacJ protein as a potential immunogen (98). Studies where vaccines made with OMVs have been used with this protein have resulted in a high titer of IgG antibodies but have not allowed effective protection against infection caused by App to be established (91, 103). Similar results were obtained with a vaccine against *Glaesserella parasuis* (104) In contrary cases, the combination of VacJ with other proteins has given 100% protection for controlling *Pasteurella multocida* (105).

In the case of the HtrA protein, it has only been possible to carry out clinical trials where it has been possible to establish that the absence of this protein in the bacteria decreases the colonization capacity, in addition to the fact that conditions of oxidative stress and high temperature potentiate infection by App (106). This result has been attributed to the fact that HtrA can cleave cell-to-cell binding factors, causing damage to the extracellular matrix, disrupting the epithelial barrier, and thus finally causing damage to the host cell. This protein is proposed as a possible antibacterial treatment or for vaccine development (100).

## Cell integrity

As previously mentioned, the cell membrane plays one of the most important roles concerning the integrity of the bacteria when it encounters the external environment with a diversity of physical, chemical, and nutritional environments. Some proteins that contribute to maintaining the cell membrane have been identified in App.

The OmlA lipoprotein is an outer membrane protein first described by Gerlach et al. (107) with a molecular weight of 40 kDa; in addition to being also found within the outer membrane vesicles (OMVs), it is detected in serotypes 1, 2, 8, 9, 11 and 12. Subsequently, allelic variations between the OmlA genes between different App serotypes were identified (108, 109). It was not until years later that App serotypes 1-15 showed a phylogeny of up to 10 variants (110). This protein has also been studied in other bacterial species such as *Pseudomonas aeruginosa, Pseudomonas fluorescens* (111), and *Xanthomonas campestris* pv*. phaseoli* (112), having a role in maintaining cell integrity, in addition to allowing iron acquisition by the host (72).

The OmlA protein has been related to the AasP protein. AasP is a 104 kDa subsistilin-like autotransporter conserved in the outer membrane, similar to a serine protease. It is experimentally transcribed in porcine lung tissue between 7 and 21 days after infection and is associated and expressed in membrane fractions. It is regulated by a global anaerobic regulator (HlyX) (113). Ali et al. (114) used recombinant AasP protein, and immunoblot analysis showed that AasP does not undergo proteolytic cleavage. Using an AasP mutant derivative and a complemented AasP mutant led to identifying OmlA fragments, suggesting that AasP is involved in OmlA modification. On the other hand, AasP is also related to other proteins of the SPATE family of serine proteases. Plasencia-Muñoz et al. (115) identified in immunoblot a band with a molecular weight of 100 kDa using a polyclonal antibody against Vat toxins, weight like the AasP protein. In a cellular interaction, the internalization of the bacteria is observed as the formation of vacuoles in endothelial cells in an *in vitro* cell model. However, it is not established whether AasP is responsible for said vacuoles.

There are other proteins related to membrane integrity. PalA lipoprotein is analogous to the OMPs associated with the PAL peptidoglycan of *E. coli*. This protein was initially purified from App serotype 2, with a molecular weight of 14 kDa, and later identified in 12 App serotypes, in addition to having a high similarity with the *E. coli* TolB protein (116). TolB interacts with other OMPs such as TolA, TolQ, and TolR and plays a role in maintaining the integrity of the outer membrane of *E. coli* (117, 118). PalA has a similarity with the P6 protein of *H. influenzae. With* an OMPs of 16 kDa of *P. multocida*, it has been shown that an antiserum against the P6 protein acts as a bactericide against clinical isolates of highly pathogenic non-typeable strains (118).

Polyamines are a group of small polycationic compounds that participate in aspects of cell physiology, stabilizing the structure of the cell membrane, in addition to participating in stress tolerance, signaling, biofilm formation, and immune system evasion. In App, the PotD periplasmic protein binds to polyamines before transport, it has been identified in 15 serotypes, and deletion of the potD2 gene can lead to defects in extracellular polyamine transport in the early period of growth, leading to cell damage (119).

The outer membrane lipoprotein VacJ in the pathogenicity of App has an important role in maintaining cell integrity and serum resistance. It has been shown that bacteria mutated with the VacJ gene show greater sensitivity to EDTA and SDS detergents, allowing greater hydrophobicity of the bacterial membrane. These same results have been seen in *S. flexneri* and *H. influenzae*. In the case of serum resistance, it has been observed that bacteria mutated with the VacJ gene are much more sensitive to porcine serum *in vitro*, reflecting the loss of the ability to resist complement factor (120).

There are few trials of vaccines with proteins involved in the integrity of the bacterial membrane. OmlA has been proposed as a potential immunogenic agent, but currently, there are no vaccines with this protein against infection caused by App (95, 121).

The only vaccine prototype for the AasP autotransporter was the study by Oldfield et al. (122). They hypothesized that the vaccine would protect pigs from infections against App, but the results were unfavorable since it did not inhibit colonization and severe clinical disease. There is no evidence of the presence of AasP in other bacterial species. However, a wide variety of other autotransporters exist in *E. coli, Citrobacter rodentium, Salmonella, Neisseria, Shigella, and Edwardsiella* species (123, 124), including the serine proteases (SPATEs) of *E. coli* the most studied, whose immunoprotective effect results have allowed us to establish that it inhibits colonization (125–128). With the results of investigations on the virulence of the group of autocarrier proteins, it has been concluded that the widespread presence of conserved and highly immunogenic epitopes suggests that they may be the target of vaccine development for infections caused by Gram-negative bacteria (128, 129).

Although in the case of the PalA lipoprotein, there are studies of vaccination in combination with other proteins, the results have not been favorable since the protective effect of different proteins decreases (118). This same result was obtained in a murine model that corroborates that in the case of this protein, the product is immunogenic but not protective (130). In the case of the PotD protein, no vaccine was developed for App, but it should be noted that there is evidence that this protein has had an immunoprotective effect against *H. parasuis* (131).

## Biofilm formation

Biofilms are structured communities of different bacterial cells within a polymeric matrix produced by themselves, which are embedded in a surface, allowing them to survive in the surrounding environment (132). It has been found that App is a bacterium capable of forming biofilms in pigs in natural infection processes (133) and that it can form multispecies biofilms with other porcine respiratory pathogens under laboratory conditions (134).

Studies of molecular factors involved in forming biofilms have focused on lipopolysaccharides (LPS) and exopolysaccharides. Still, there are also important studies concerning the role played by proteins in the adhesion and formation of biofilms. This close relationship between these last two virulence factors could be vital to understanding the pathogenicity of App (133, 134).

Tegetmeyer et al. (2) found that the AasP autotransporter does not have adhesion properties. Still, they propose that it participates in the biosynthesis and degradation of poly-N-acetyl-glucosamine (PGA), which are the main and indispensable components for the formation of the biofilm matrix of App. Xie et al. used a VacJ lipoprotein mutant, resulting in poor *in vitro* biofilm formation at 36 h compared to the wild-type strain (120).

Assays with mutants and gene deletion have been used to evaluate the role of specific proteins in biofilm formation, the results of which have detected the participation of surface proteins (48, 135–138) (Table 2).

**Table 2:** Proteins involved in biofilm formation of *Actinobacillus pleuropneumoniae.*

Bacterial isolate characteristics	Protein	Gen	Effect on biofilm	Reference
Mutants App 1 (4074)	APL_0049e: hypothetical protein DNA-binding protein 2 Hypothetical proteins APL_0650: Hypothetical protein APL_0651: UTP-glucose-1-phosphate uridylyltransferase	hns galU	Production enhanced biofilm	Grasteau et al. (135)
Mutants App 1 (4074)	Ribonucleoside-diphosphate reductase large chain tRNA-dihydrouridine synthase B Spermidine/putrescine-binding periplasmic protein APL_0384e: riboflavin biosynthesis protein Orotidine 50-phosphate decarboxylase Phosphoenolpyruvate-protein phosphotransferase 50S ribosomal protein L32 Trigger factor APL_1572e: Hypothetical protein	rnr1 dus potD2 ribAe pyrF ptsI rpmF tig	Production enhanced-biofilm reduced-biofilm	Grasteau et al. (135)
S8ΔclpP, clpP mutant App 7 (S8)	ClpP	clpP	Poor biofilm formation	Xie et al. (136)
5bØAdh, adh deletion mutant of App 5b (L20)	Adh	adh	Weak ability to form biofilms on tubular and flat surfaces	Wang et al. (48)
TolC1::cat, tolC mutant App 7 (SC1516)	TolC1	tolC1	Decreased biofilm formation	Li et al. (13)
△lonA and △lonC. lonA and lonC mutant App LD20	LonA LonC	lonA lonC	Poor biofilm formation Slightly decreases biofilm formation	Xie et al. (137)

Although there is very little information about vaccines made with biofilm-related immunogens, some proposals suggest that they could effectively prevent and control App infections. Still, more studies are required to identify relevant molecules expressed in biofilms during infection (139).

## *Actinobacillus pleuropneumoniae* proteome

To understand the molecular interactions a little better, it has been decided to carry out proteomic analyses of the cell surface. Proteomic studies, in addition to identifying many proteins, also reveal information about their internal and external structures, their composition, mechanisms of action including biofilm formation, antimicrobial resistance, and their way of adapting to the external environment (140).

Over the last decades, advances have been made that allow a better understanding of the pathobiology of the infection, and genetic tools have also allowed a better understanding of the molecular mechanisms of App. However, despite this, many of the molecular functions of this bacterium are still unknown (27).

Regarding the identification of OMPS of the bacterium App. Chung et al. (141) identified 47 OMPS in serotype 5b, representing 50% of the proteome and finding that the proteome is mostly hydrophilic. Liao et al. (30) identified 30 proteins for serotype 3, where 6 antigens were already known (MomP1, MomP2, ApxIIA, ApxIIIA, NqrA, and FhuA), and 24 more were identified for the first time. Subsequently, Zhang et al. (108) conducted a genomic sequencing analysis for serotype 1, where they identified 32 total proteins (two of them being outer membrane protein protective surface antigen D15 and outer membrane proteins P5). Chung et al. (109) also developed a work to identify new App antigens through the outer membrane proteome and under iron restriction conditions; in this work, they were able to identify the proteins TbpA, TbpB, PotD2, HbpA, CpxD, and OmlA.

Zhu et al. (91) carried out a study of the OMVs proteome and determined the content within them where they found 82 proteins whose origin is mainly from the outer membrane, periplasmatic proteins, and extracellular toxins (MomP2, OmlA, FkpA, OmpP1, LpoA, and ApxIII extracellular toxin), they also found other proteins that are involved with the absorption and restriction of iron (HbpA1, TbpB1, FrpB, HbpA2, YfeA, AfuA2, FhuA, TbpA) and a periplasmic protein of Fe type ABC^3+^. It has been shown that some proteins, such as PalA and VacJ, increase the secretion of OMVs (96–98).

Many proteins can also be secreted into the external environment of the bacterium. Stancheva et al. (142) identified 593 exoproteins, of which 104, through a prediction study, were proposed to be important virulence factors. Among the most found proteins were the Apx toxins.

**Figure 2:**
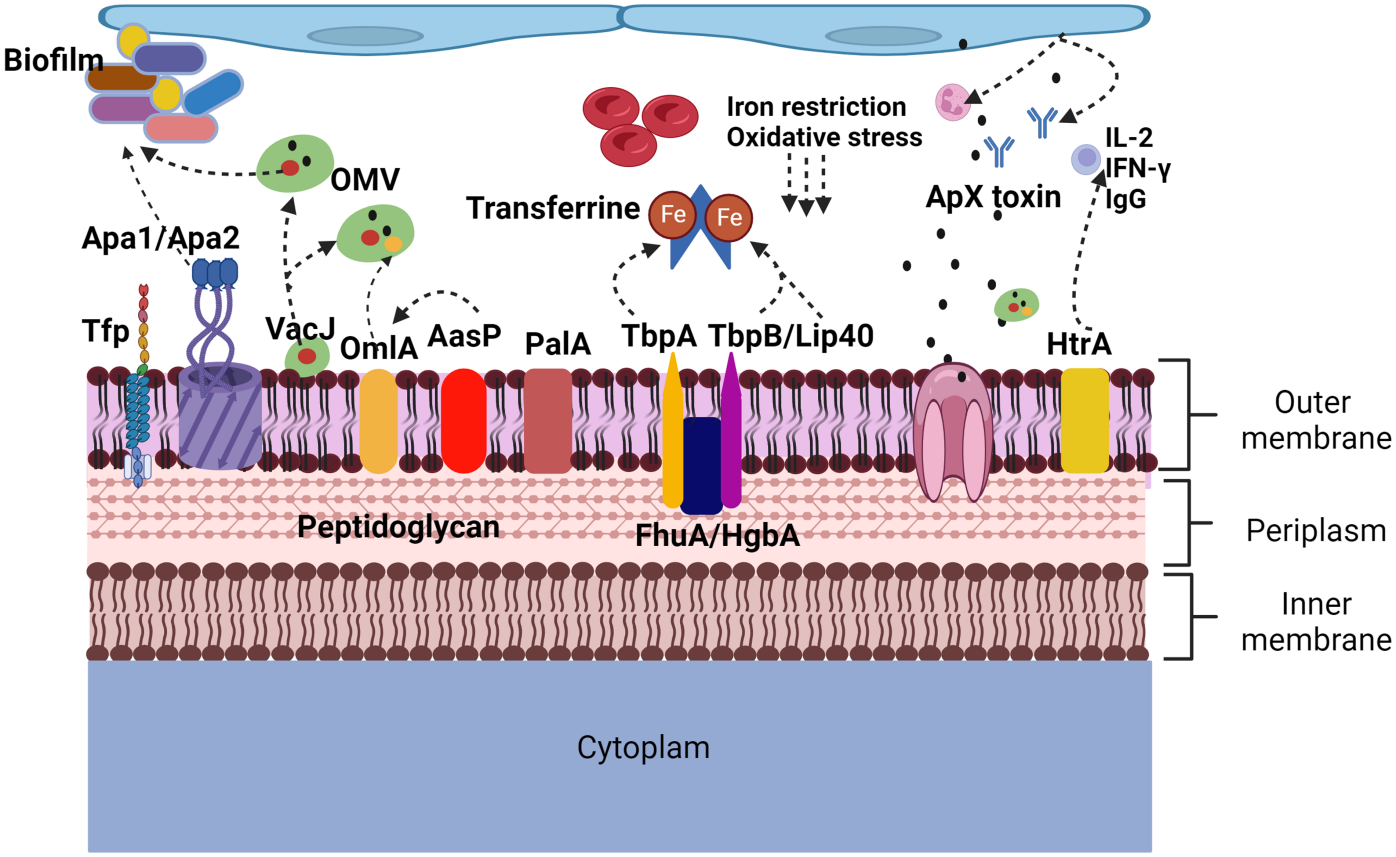
Proteins involved in virulence factors of Actinobacillus pleuropneumoniae. App adherence is regulated by lipopolysaccharides but also by proteins such as ApfA adhesins that allow adherence to collagen and fibrinogen, Apa1 and Apa2 that, like type IV Tfp pili, allow biofilm formation. In the induction of lesions and evasion of defense mechanisms, the Apx toxins cause neutralization of antibodies, lysis of neutrophils and red blood cells, the HtrA protein induces the secretion of IgG, IL-2 and IFN-γ. The OmlA protein plays an important role in the cell integrity of bacteria, in addition to being found within the vesicles of the outer membrane, VacJ, in addition to being found within the vesicles of the outer membrane, increases their secretion, it also participates in the formation of biofilms and plays an important role. Important role in serum resistance. PalA binds peptidoglycan to the outer membrane. AasP modifies the secretion of OmlA. The TbpA, TbpB, Lip40 and FhuA proteins are related to the iron acquisition virulence factor, are upregulated under anaerobic conditions and oxidative stress.

The large number of proteins currently identified (Figure 2) opens a window of opportunity to increase knowledge about the role they play individually, how they could be related to each other at a molecular level, and how they participate in virulence factors, being able to propose them as possible immunogens for the elaboration of future vaccines.

## Potential vaccines

A wide variety of vaccines have contributed to reducing mortality and morbidity caused by App in pig farms. In the review work carried out by Loera et al. (95) we found that there is an excellent diversity of live attenuated and inactivated vaccines made with Apx toxins, membrane proteins, and combinations of these, DNA-based vaccines, which provide a protective effect in pigs and mice, but the search for new alternatives continues.

Highlighting the importance that proteins have acquired as potential immunogens for the preparation of future vaccines, the existence of moonlighting proteins has also been found. These proteins are expressed by the same gene and perform two or more functions in an organism in addition to having molecular links in different biological processes, whose role in bacteria is to participate as chaperones in response to stress and their ability to participate in pathogenesis as a factor of virulence once it is secreted into the external environment (143, 144).

The participation of these proteins in virulence factors focuses on adhesion to epithelial cells, especially to mucus and components of the ECM. One of the moonlighting proteins that have been found most frequently is the GAPDH protein; in *Streptococcus pyogenes,* it participates in the binding to cellular components such as plasmin, actin, myosin, and fibronectin; in *Streptococcus pneumoniae,* it also engages in the imperative to plasmin, in *Streptococcus agalactiae* functions as a stimulator of B lymphocyte activity, in *Bacillus anthracis* it functions as a plasminogen-binding protein. It may have NAD-ribosylating activity in enteropathogenic *E. coli* and enterohemorrhagic *E. coli*. Other moonlight proteins found in bacteria that also have similar activities are Enolase, 17 glycolytic enzymes, FbaA, Pgk, Eno, EF-Tu, PdhB, malate synthase, SodA, DnaK, GroEL, Tig, TP1 and GS, where their secretion and localization are regulated by specific stimuli (144).

In a study by Stancheva et al. (142) in a proteomic analysis in App serotype 2, moonlighting proteins were found, highlighting EF-Tu as a potential immunogen for new therapies and vaccines. However, there are still no studies that evaluate its effectiveness. These proteins have been considered as candidates for vaccines against fungi, such as the Fba1, Pgk, and Pk proteins of *Candida albicans* and *Candida glabrata*, conferring protection in a murine model, although *in vivo* studies are still lacking (145). A prototype vaccine with the moonlighting protein Fba (a protein with a central position in the glycolytic pathway) has resulted in a higher survival status in fish and a decrease in bacterial concentration in the spleen of the bacterium *Photobacterium damselae* subsp. *piscicide*, considered one of the most dangerous pathogens in aquaculture (146).

In general, many membrane proteins, pili, OMPs, and OMVs, among others, have been proposed as potential candidates for the preparation of future vaccines. Their participation in virulence factors has been studied. However, there is still a need to design and apply vaccines *in vivo* models to determine their effectiveness against infections against App.

## Isolation and identification of proteins

An essential step in carrying out studies on the pathogenesis of infectious agents is to develop protocols that allow the isolation and identification of immunogens, where molecular factors, specifically surface proteins, play an important role.

As previously mentioned, the bacterial membrane has many components, so it is sometimes easy for samples to be contaminated with cellular by-products during protein extraction or for proteins to be easily denatured during the experiment (147). Therefore, it is important to be clear about the target proteins to be isolated to handle the samples properly.

Cell lysing methods used for subsequent protein identification include physical processes, such as heat treatment, sonication, osmotic shock, and chemical methods, including treatment with a detergent solution (EDTA), polymyxin, and extraction with chloroform. These techniques allow the recovery of up to 90% of specific proteins (147, 148).

Most of the protein extractions in the App use sodium lauroyl sarcosinate (Sarkosyl). This detergent has been shown to have a milder effect than sodium dodecyl sulfate (SDS) and can identify proteins with resistance to SDS (149). Trichloroacetic acid (TCA) can be used to search for target proteins through isoelectric precipitation and solvents such as methanol and acetone to promote the precipitation of said proteins (147). TCA is mainly used in proteomic studies to search for isoelectric points in 2D gels where proteins must be free of contaminating residues (150). These last two techniques used in the reviewed studies identify many proteins found on the surface of the App bacterium (21, 28–30) (Table 3).

**Table 3:** Immunoprotein analysis of *Actinobacillus pleuropneumoniae*.

Serotype/Strain App	Protocols for protein extraction	Identified proteins	Reference
Serotype 5b (L20)	Sarkosyl-insoluble membrane proteins Pellet suspension of bacterial cells in PBS, lysis buffer in Tris-sarkosyl Recovery of OM vesicles by centrifugation Suspension with distilled water Cold acetone wash for protein precipitation Suspension of proteins in water. Outer membrane vesicle proteins by SDG SDG is performed on pelleted total membranes in HEPES lysis buffer Ultracentrifugation Dilution of protein fractions in Tris–HCl Centrifugation Washes in Tris-sarkosyl buffer, sodium carbonate, sodium bromide with calcium carbonate or NBSC treatment Wash and suspension in Tris–HCl	OMP P2, PAL cross-acting lipoprotein, uncharacterized protein HI0449; TbpA, Uncharacterized protein HI1462, Hemin receptor, Heme/hemopexin utilization protein C, OMP P6, uncharacterized protein PM1805, SPBP, Glycerophosphoryl diester phosphodiesterase periplasmic, OMP P4, OM protein D15, Outer membrane protein assembly factor BamE, OMP P5, LolB, Mlt38, TamA, SrpC. HI0922, PLP1, 47 kDa outer membrane protein, LptD, Probable hemoglobin and hemoglobin-haptoglobin-binding protein 4, Outer membrane protein W, Outer membrane protein assembly factor BamD, PBP activador LpoA, OMP P5, TbpB, Capsule polysaccharide export protein BexD, 24 kDa outer membrane protein, OMP P5, Endopeptidase O, VacJ, Outer membrane antigenic lipoprotein B, Ferric hydroxamate receptor, Lipoprotein nlpE, PPIase, OmlA, LppB, two proteins not found	Chung et al. (141)
Serotype 3 (JL03)	Extracellular proteins: Protein precipitation from the supernatant with TCA Obtaining precipitate by centrifugation Wash with cold acetone and DDT Speed vacuum treatment Solubilization of proteins with urea, thiourea, CHAMPS and DDT Outer membrane proteins: Obtaining and washing bacterin cell pellet suspension with Tris-EDTA Sonication Centrifugation and dilution with Na_2_CO_3_ OMPs granulation by ultracentrifugation Resuspension of pellet with Tris-EDTA Centrifugation and solubilization with lysis buffer	MomP1, MomP2, ApxIIA, ApxIIIA, NqrA, FhuA, D15 / OmpD, LppB, FrdA, MDH, FepA, FrpB, TufB, PotD, GapA, ZnuA, TIG, DegP, TufB, PsaA, FkpA, PTA, CbiK, IlvG, FepB, AfuC, FatB, GGBP, CysG y Ttg2D	Liao et al. (30)
Serotype 1 (S259)	Immunoprotein analysis Suspension of the bacterial cell pellet in urea, thiourea, CHAMS, and DTT Sonication Incubation for protein solubilization Centrifugation of insoluble components Protein precipitation with cold TCA Incubation and washing with cold acetone Air-dried pellet	Polyribonucleotide nucleotidyltransferase, ATP-binding protein, Chaperonin GroEL (HSP60 family), CTP synthetase, Chaperone protein dnaK, fumarate hydratase, bifunctional UDP-sugar hydrolase/5’-nucleotidase periplasmic precursor, Transketolase 2, Translation elongation factors (GTPases), TPR repeat, Sugar transferases involved in lipopolysaccharide synthesis, hypothetical protein appser1_10340, ype I restriction enzyme EcoEI R protein, GTP-binding protein typA/bipA, hypothetical protein appser1_12060, Asparagine synthetase A, BioD-like N-terminal domain of phosphotransacetylase, protective surface antigen D15 precursor, Protective surface antigen D15, DNA topoisomerase III, ApxIIA, Outer membrane protein P5, ABC-type transport system involved in resistance to organic solvents, auxiliary component, Periplasmic component of the Tol biopolymer transport system, Ribosomal protein L7/L12, putative aldehyde dehydrogenase, pyruvate dehydrogenase subunit E1, Na + -transporting NADH:ubiquinone oxidoreductase, subunit NqrA, outer membrane protein P5 precursor, Outer membrane protein and related peptidoglycan-associated (lipo)proteins	Zhang et al. (108)
Serotype 5 (L20)	Immunoprotein analysis of membrane proteins under iron-restricted growth conditions Addition of EDDHA in logarithmic phase (OD600 0.1) of App growth Bacterial lysate by French pressure Isolation of outer membrane vesicles by sucrose gradients Membrane washes with sodium bromide and sodium carbonate were previously described (141) Suspension in Tris–HCl	TbpA, TbpB, spermidine/putrescine, PotD2, HbpA, CpxD, OmlA.	Chung et al. (109)

Since proteins cannot be chemically synthesized, it was decided to elaborate them by biological processes with other bacteria (151), by obtaining recombinant proteins, or by mutants where the deletion or insertion of protein genes to be studied is carried out. These techniques have also been widely used, allowing the screening of specific proteins and the antigenic enrichment of the outer membrane of App (141, 152) as well as the isolation of OMVs (98) (Table 4).

**Table 4:** Outer membrane enrichment and obtaining outer membrane vesicles for the identification of *Actinobacillus pleuropneumoniae* proteins.

Serotype/Strain App	Protocols for protein extraction	Identified proteins	Reference
App MIDG2331 wt, ΔdegS, and ΔnlpI clones	Obtaining outer membrane vesicles Filtering of supernatants of bacterial cultures Hydrostatic Filtration (HF) Filtering and suspension of concentrated supernatant Dialyzate in sterile PBS Concentration of outer membrane vesicles at (200-fold concentration)	VacJ y ApfA	Antenucci et al. (98)
Serotype 8 (MIDG2331), pMK_apfas-ACPm-vacJ transformed	Outer membrane enrichment Collection of bacterial cells Wash with HEPES and storage at-80°C Thawing, lysis with DNase and lysozyme using a bead beater Centrifugation to remove cell debris Centrifugation of supernatants for recovery of cell membranes Resuspect with HEPES Solubilization with Sarkosyl in HEPES Granulation by centrifugation Protein resuspension with HEPES	VacJ	Antenucci et al. (152)

To carry out said protein isolations, knowing under what growth conditions they are expressed is essential. It has been shown that some proteins are expressed in a greater or lesser proportion depending on the bacterial growth conditions in which they are immersed. In App, a difference in protein expression was found under normal growth conditions (153) compared to proteins obtained under nutrient addition conditions such as maltose (57), anaerobiosis, and different temperatures (154), as well as the restriction of iron (64) (Table 5). Protein expression under these growth conditions has also been tested in other bacteria, such as *Salmonella typhimurium* (155), *E. coli* (156), and *Pasteurella haemolytica* (157). Knowing how this bacterium behaves molecularly under stress conditions can help to find possible routes to improve management and treatments in App.

**Table 5:** Obtaining *Actinobacillus pleuropneumoniae* proteins under different bacterial growth conditions.

Serotype/Strain App	Protocols for protein extraction	Identified proteins	Reference
Serotype 1 (4074), Serotype 5 (1A, 155, 163, 178, 200, 201), Serotype 7 (WF83)	Protein identification under natural infection conditions Bacterial Pellet Suspension in HEPES Sonication Centrifugation to remove insoluble components Centrifugation for sedimentation of membranes Solubilization of membranes with sarkosyl Sedimentation of OMPs-enriched fractions by centrifugation Protein suspension is deionized water.	16.5 K, 29 K, 38.5 K, 43.5 K, 45 K, 49.5 K, 66.5 K	Rapp et al. (153)
Serotype 1 (79-9) Clinical isolates Serotype 1 to 7	App culture media with maltose addition Routine cultivation of App with the addition of maltose at 0.4% (w/v) Obtaining OMPs-enriched fractions Washing of bacterial pellet with HEPES buffer Sonication Centrifugation of insoluble components Incubation for 10 min with sarkosyl Centrifugation for sedimentation of proteins Resuspension with HEPES and sarkosyl	42 KDa protein	Deneer et al. (57)
Serotype 1 (SLWO1), Serotype 10 (13039)	Culture media in anaerobiosis at temperatures of 42, 16, and 37° C Obtaining bacterial pellet and resuspension in Tris–HCl, Sucrose, and EDTA Centrifugation to remove insoluble components Resuspension of pellet in cold Tris–HCl, EDTA, DTT, and DNase Centrifugation Separation of cytoplasmic fraction and crude membranes pellet Resuspension of the raw membrane pellet in Tris–HCl, Triton, and MgCl_2_, Centrifugation Washing of outer membranes in Tris–HCl, Triton, and MgCl_2_, Three washes with dH2O Filtration of extracellular proteins in trichloroacetic acid Centrifugation Six washes with 96% ethanol Drying and resuspension with urea, thiourea, CHAPS, Tris–HCl.	Lip40	Hu et al. (154)
Serotype 1 (4074), Serotype 2 (4226), Serotipo 3 (1421), Serotype 4 (1462), Serotype 5 (K-17), Serotype 6 (FEMO), Serotype 7 (WF83)	Culture media under iron restriction Addition of EDDHA from the beginning of the inoculation of the bacteria in routine culture medium Obtaining bacterial pellet Sonication Centrifugation Resuspension in water Dilution in Tris-Sarkosyl Ultracentrifugation for recovery of OM vesicles	HgbA	Srikumar et al. (64)

## Conclusion

There is a great diversity of proteins that are not only secreted by the App, but also form part of the bacterial surface and actively participate during infection. The Tfp, ApfA, Adh, and Apa2 proteins participate in adhesion. A 42 kDa protein is actively expressed in the presence of maltose. FhuA, FhuB, FhuC, FhuD, and HgbA are proteins related to the iron acquisition factor. LamB is the porin-associated protein involved in drug resistance. The ApxI, ApxII, ApxIII, and ApxIV toxins are the proteins most related to the production of lesions and stimulation of the immune system. However, other proteins, such as VacJ and HtrA, have also been found to cause lesions. Proteins such as OmlA, PalA, and PotD participate in the maintenance of cell integrity. The AasP protein, in addition to participating in the modification of OmlA, is also related to the biosynthesis of other adhesion factors in the formation of biofilms; in the same way, VacJ engages in the maintenance of cell integrity, these last two proteins are related in more of a virulence factor. Many proteins have only been identified, but their role in the infection caused by App has not been established. It is essential to continue the search for new immunogens that allow the establishment of an effective vaccine against all App serotypes. Using computer tools that contain App proteomics information has permitted the development of reverse or *in silico* vaccinology techniques, finding new immunogens focused on surface proteins. In this study, we found that combining different surface proteins could be the key to developing an effective vaccine against App, although *in vitro* and *in vivo* studies are necessary to establish the efficacy of these vaccines.

## Author contributions

MMSP: Investigation, Methodology, Writing – original draft. ALGB: Conceptualization, Formal analysis, Funding acquisition, Resources, Writing – review & editing, Project administration. FJAG: Writing – review & editing. TQT: Writing – review & editing. OMM: Writing – review & editing.
